# The ‘Operational’ Definition of Self-Control

**DOI:** 10.3389/fpsyg.2018.01231

**Published:** 2018-07-18

**Authors:** Marleen Gillebaart

**Affiliations:** Social, Health, and Organizational Psychology, Utrecht University, Utrecht, Netherlands

**Keywords:** self-control, self-regulation, initiation, inhibition, TOTE-model, effortful control

## Abstract

Self-control is a hot topic across disciplines. As such, consensus on defining self-control is critical for advancing both scientific progress as well as societal impact of research findings. Specifically, the emergence of initiation as a self-control component, and the notion of effortless and strategic self-control, give rise to the question whether and how to distinguish self-control from self-regulation. In this paper, I propose an operational definition of self-control, based on converging definitions from the literature as well as on the emergence of new perspectives on self-control. The TOTE-model (Test-Operate-Test-Exit) of self-regulation will serve as a basis for this definition as it gives clear guidance for the inclusion of self-control as a component of, but not synonymous to self-regulation. Ultimately, an ‘operational’ definition is proposed in which self-regulation entails scaffolding for goal pursuit, including setting standards, and monitoring discrepancies, whereas self-control entails everything that one does in the ‘operate’ phase. This perspective allows for inclusion of traditional as well as contemporary research on self-control, and can provide direction for future studies.

## Introduction

Self-control is a hot topic across disciplines. Scholars from social, health, and personality psychology, as well as from developmental and brain sciences, to name a few areas, devote their work to understanding the causes, consequences, and underpinnings of this key human trait. As such, consensus on what we mean when we use the term self-control is critical. Without such consensus, synthesizing research on self-control is precluded, hindering both scientific progress as well as societal impact of research findings. However, recent developments in self-control research seem to have muddled the definition waters, causing some confusion about what self-control entails, and what it does not. Specifically, the emergence of initiation as a self-control component, and the notion of effortless and strategic self-control, give rise to the question whether and how to distinguish self-control from self-regulation. In this paper, I propose an operational definition of self-control, based on converging definitions from the literature as well as on the emergence of new perspectives on self-control. The TOTE-model (Test-Operate-Test-Exit, Carver and Scheier, 1982) of self-regulation will serve as a basis for this definition as it gives clear guidance for the inclusion of self-control as a component of, but not synonymous to self-regulation.

The importance of self-control for behavior and well-being is undisputed. Several studies have shown that self-control level at a young age can predict cognitive and self-regulatory skills in adolescence (Shoda et al., 1990), as well as essential outcomes such as health and well-being later in life (Moffitt et al., 2011). Moreover, having self-control is related to better grades and academic achievements (Tangney et al., 2004; Duckworth and Seligman, 2005), better quality interpersonal relationships (Vohs et al., 2011), and basically, a happier life (Cheung et al., 2014; Hofmann et al., 2014). Conversely, being prone to low self-control is associated with problematic behaviors and outcomes such as impulse buying (Baumeister, 2002) and financial debt (Gathergood, 2012), maladaptive eating patterns (Elfhag and Morey, 2008), and procrastination (Tice and Baumeister, 1997). Because of these robust associations between self-control and this myriad of behaviors and outcomes, self-control has been coined a ‘hallmark of adaptation’ (De Ridder et al., 2012).

For such an essential psychological construct, the dispersion of definitions is remarkable to say the least (see also Milyavskaya et al., 2018). For example, in terms of operationalizations, the amount of self-control measures easily reaches a 100 (Duckworth and Kern, 2011). Before integrating perspectives on self-control, let us first discuss the most prominent definitions that are out there already. One of the narrower definitions of self-control equates the concept with inhibitory control. In this definition, self-control includes, and is limited to, the effortful inhibition of impulses. This inhibition is the key self-control component in many self-control theories and models, including those based on delay of gratification (Ainslie, 1975; Mischel et al., 1989; Kirby and Herrnstein, 1995) and dual-systems frameworks (e.g., Metcalfe and Mischel, 1999; Hofmann et al., 2009). Dual-systems theories are characterized by the notion of two systems for processing information and guiding behavior. The ‘hot’ system is fast, associative, continuously ‘on,’ and provides impulsive tendencies for behavior. The ‘cold’ system on the other hand is a bit slower, can only function when enough resources (e.g., energy, attention) are available, and is more likely to initiate rationalized behavior (Evans, 2008; Kahneman, 2011). Self-control can, according to this perspective, be defined as the mechanism that allows for inhibiting or overriding impulses coming from the hot system, allowing precedence of the cold system (Gillebaart and De Ridder, 2017).

Self-control has also been defined as the ability to delay immediate gratification of a smaller reward for a larger reward later in time (Ainslie, 1975; Mischel et al., 1989; Kirby and Herrnstein, 1995). This definition includes the effortful inhibition notion, but is extended in the sense that it emphasizes the self-control dilemma or conflict between a short-term, immediately gratifying option (that needs to be inhibited) and a long-term option with a larger reward value. The ability to forego the immediate reward reflects self-control.

A related model of self-control is the strength model of self-control (Baumeister and Heatherton, 1996; Muraven and Baumeister, 2000). The strength model is one of the most prominent, heavily debated models of self-control, and refers to self-control as ‘... an act of self-control by which the self alters its own behavioral patterns so as to prevent or inhibit its dominant response’ (Muraven and Baumeister, 2000, p. 247). The most significant proposition from this model entails the ‘ego depletion’ phenomenon. Based on the models tenet that self-control is effortful, ego depletion describes the self-control failure that can follow from earlier effortful self-control exertion due to depletion of a limited self-control resource. Importantly however, this model focuses on state self-control, precluding a broader perspective on self-control as a disposition or trait.

These traditional definitions of self-control have two key aspects in common: effort and inhibition. However, over the past decade several researchers have suggested and shown that in order to be able to successfully use self-control in daily life, one needs to do more than simply effortfully inhibit impulses and unwanted responses in specific instances. With regards to inhibition, many long-term goals of course do require inhibition of responses that are in correspondence with short-term goals, but not with long-term goals. For example, one may have a long-term goal to have a healthy body, and may therefore need to inhibit the urge to bury one’s face in chocolate cake. Or, one may want to achieve academic success, and may therefore need to inhibit the binge-watching impulse fed by the Netflix algorithm. However, these long-term goals of a healthy body and academic success are not achieved by solely inhibiting impulsive behaviors that are incongruent with long-term goal pursuit. In fact, initiation of long-term congruent behaviors may be equally, if not more important. For example, to have a healthy body in the long run, one needs to initiate the consumption of healthy foods like fruits and vegetables on a regular basis. Likewise, to be successful in terms of academic performance, one needs to initiate a lot of behaviors that may not be immediately satisfying (and sometimes even no fun at all). Indeed, De Ridder et al. (2011) were able to define both an inhibitory and an initiatory component of self-control, with inhibitory self-control predicting undesired behavior, and initiatory self-control predicting desired behavior. Acknowledging initiation as a component of self-control holds implications for self-control’s definition, and may mean that this definition needs to be updated to align with these current insights.

Following the acknowledgment of initiation as an essential part of self-control, it was proposed that self-control can be conceptualized as the resolution of the conflict between two motives (i.e., short-term and long-term), with emphasis on the notion that effortful inhibition is but one of many possible ways of handling these types of dilemmas (e.g., Fujita, 2011; De Ridder et al., 2012). Taking it even further, Gillebaart and De Ridder (2015) suggest that self-control simply *cannot* rely on effortful inhibition only, because this would render people extremely prone to self-control failure all the time, due to depletion, fatigue, or lack of attentional or motivational resources. In reality however, many people succeed in using their self-control in subsequent situations. Gillebaart and De Ridder suggest that people who have a high level of (trait) self-control generally do not actually use effortful inhibition to resolve self-control dilemmas, but instead use their self-control to install ‘smart,’ relatively effortless strategies for long-term goal-congruent behaviors.

One of these proposed self-control strategies is the automatization of adaptive behaviors. Recent studies have supported this proposition by showing that people with higher levels of trait self-control have habits that align with their long-term goals. People with high trait self-control have stronger habits for studying and healthy eating (Galla and Duckworth, 2015), as well as for exercising (Gillebaart and Adriaanse, 2017). Interestingly, higher self-control does not necessarily mean stronger habits across the board. A study by Adriaanse et al. (2014) demonstrated that people with higher levels of self-control in fact have a weaker habit for eating unhealthy snacks. The important conclusion from these studies is therefore not that people with high self-control have stronger habits, but rather that their response to environmental cues is automatized in the direction that is in line with their long-term goals. This allows for an effortless way of resolving self-control dilemmas. A meta-analysis on the association between self-control and a range of behaviors supports this notion by demonstrating stronger effects of self-control on automatic behaviors than on deliberate behaviors (De Ridder et al., 2012). Taking automatic self-control behaviors into account, the notion of ‘effort’ that has also been central when defining self-control needs to be revisited.

Further research into effortless self-control strategies has indicated that people with high self-control use their self-control to create environments for themselves that are in congruence with their long-term goals. An example of such a strategy is pro-active avoidance (Ent et al., 2015; Gillebaart and De Ridder, 2015). People with higher levels of self-control initiated behavior aimed at avoiding temptations, and when given the option more often chose to work in an environment void of distraction (Ent et al., 2015). Avoiding a temptation at an early stage allows for relatively effort-free self-control, as regulation of an impulsive state becomes more difficult as this state unfolds over time (Gross, 2014). Avoiding temptations, and thus self-control dilemmas, thus leads to less need to use effortful self-control (i.e., effortful inhibition of impulsive tendencies). This is reflected in daily life as well, as a diary study on self-control and daily experiences of desire, temptation, and conflict demonstrated that higher self-control was associated with fewer experienced temptations, and fewer instances of self-control conflict and resisting temptations (Hofmann et al., 2012). Moreover, if people with high self-control do encounter self-control dilemmas, they are able to resolve those dilemmas in a more efficient way compared to their low self-control counterparts (Gillebaart et al., 2016). Taken together, research shows that there are different strategies for self-control, differing in how much effort they cost, whether they focus on inhibition or initiation, how automatized they are, and where they are applied on the self-control dilemma timeline.

These recent studies on self-control and automatic, habitual and strategic self-control behaviors further emphasize the need to have a good look at the definition of self-control as being effortful, and focused on inhibition. In fact, considering these new developments in the field of self-control, the self-control definition is in desperate need of an update. However, including initiatory self-control and effortless self-control in the definition of self-control does pose a theoretical question: to what extent are we still talking about self-control, and to what extent are we talking about the more broadly defined concept of self-regulation? One may argue that we can hold on to our classic definition(s) of self-control, by simply stating that the self-control strategies that include initiation, smart use of strategies, and do not rely on effort, are actually not self-control strategies, but rather are part of what we call ‘self-regulation.’ Self-regulation can be defined as the whole system of standards, thoughts, processes, and actions that guide people’s behavior toward desired end states (Carver and Scheier, 2012). These desired end states may be long-term goals, but can also refer to other standards or norms. From this definition it is obvious that self-regulation and self-control are closely related concepts. In fact, they can be become so intertwined, that the terms are being used interchangeably. The distinction between self-regulation and self-control can apparently be so complex, that in the same line of research, the distinction is sometimes explicitly made (e.g., Baumeister and Vohs, 2003), where other times the two terms are seemingly treated as referring to the same thing (e.g., Baumeister et al., 2007). However, lumping the two terms together as if they are the same thing does not do either of the concepts justice.

I propose that the terminological and theoretical dispute between self-regulation and self-control that follows from recent developments in research on self-control processes can be resolved by referring back to fundamental theoretical frameworks of self-regulation that include feedback loops, such as the cybernetic TOTE model (Powers, 1973). Carver and Scheier (1981, 1982) identified three main ingredients of self-regulation: standards, monitoring, and operating. In order to self-regulate successfully, there needs to be some sort of desired end state or standard that is identified by the individual. Without such a standard, there is no direction for self-regulation, and also no motivation to steer or alter any behavior in a specific direction. In order to apply self-regulatory effort, an individual needs to be able to monitor any discrepancies between the current state and the standard (‘Test’), as well as any progress that is taking place. Finally, one needs to be able to actually control behavior into the desired direction (‘Operate’). The result serves as input for the second ‘Test’ phase. The feedback loop is exited if the current state is in line with the desired state or standard. Importantly, both setting standards or goals, and monitoring any discrepancies are part of this self-regulation feedback loop. Self-regulation therefore involves much more than simply controlling behavior, but rather provides the entire scaffolding for successful goal pursuit.

The crucial self-control element within the self-regulation feedback loop is ‘Operate.’ The difference between self-regulation and self-control therefore is that self-regulation ability allows people to formulate goals, standards, and desired end-states, as well as to monitor any discrepancies between one’s current state and these desired end-states, whereas everything that one *does* to steer one’s behavior toward the desired end state constitutes self-control. Phrased differently, everything that takes place in Carver and Scheier’s ‘Operate’ phase is what we would call self-control. Although this distinction or categorization has been alluded to before (e.g., Baumeister and Vohs, 2003), and shares some aspects with a recent analysis of self-control as being a value-based choice (Berkman et al., 2017), its importance for the current developments in the field has not been acknowledged up until now.

This ‘operational’ definition of self-control in itself may not be new, but it does emerge anew from the current developments in the field that shift away from classic theories and definitions. At the same time, it also departs from these definitions, leaving room for a new perspective. Specifically, taking this perspective on the concept of self-control allows for inclusion of classic as well as contemporary work on self-control. It also goes beyond the phenomenon of ego depletion, and allows for including state self-control as well as the more dispositional trait self-control, which is in fact predictive of many positive and negative life outcomes (Tangney et al., 2004). Moreover, ‘operate’ may have been identified as the self-control component of self-regulation, yet what is meant by ‘operate’ is in need of clarification and specification. As stated earlier, in the current perspective I propose that everything that one does to adjust one’s behavior toward a desired end state is part of ‘operate,’ and therefore part of self-control. This means that effortful as well as effortless self-control, inhibition as well as initiation, and deliberate as well as automatic actions can all be included into this definition of self-control, without convoluting the distinction between self-control and self-regulation. For example, suppressing one’s impulses to give into temptations that are not in line with our long-term goals (i.e., desired end states) is ‘operating,’ and so is inhibiting an unwanted response. Similarly, habitually avoiding the candy isle in the supermarket falls under ‘operating’ in order to reach a long-term goal of staying healthy. Likewise, being able to delay gratification by an instant, smaller reward in order to receive a larger delayed reward is ‘operating’ in terms of the self-regulation feedback loop.

## Concluding Remarks

The most interesting consequence of this conceptualization of self-control is the fact that defining self-control as the set of skills, capacities, and behaviors that we need to ‘operate’ in a self-regulation feedback loop allows for inclusion of the recently identified ‘smart’ or ‘effortless’ strategies that people with high self-control seem to (successfully) use. Importantly, this definition does not exclude the more narrow or classic definitions of self-control that have focused on effort and inhibition, but rather allows for a broader perspective that integrates these different aspects. A sidenote that comes with this analysis is that there may be situations in which monitoring itself may become a self-control issue (e.g., when one anticipates large discrepancies with the goal). In these cases, a second feedback loop for the subgoal of monitoring one’s current state is created. In these instances, the operating phase (and therefore self-control) may also refer to the action of monitoring.

The ‘operational’ definition of self-control also allows for a new line of empirical questions, answering of which would deepen our knowledge of self-control. For example, self-control strategies, whether it is inhibition or initiation, smart or effortful in nature, automatic or deliberate, all guide behavior and are ‘stored’ in the operate phase. However, one may wonder whether there are preferred strategies in general, or differing per individual, or per situation. A certain ‘ranking’ of self-control strategies is not implausible, as some require fewer resources than others (e.g., habits vs. effortful inhibition), and the timeline of a self-control dilemma affects what type of strategy is needed to resolve it Duckworth et al. (2016). Redefining self-control with a fresh perspective therefore allows research into self-control success, and smart self-control strategies, to flourish and ultimately advances the field.

## Author Contributions

The author confirms being the sole contributor of this work and approved it for publication.

## Conflict of Interest Statement

The author declares that the research was conducted in the absence of any commercial or financial relationships that could be construed as a potential conflict of interest.
